# Evaluation of NAVA-PAP in premature neonates with apnea of prematurity: minimal backup ventilation and clinically significant events

**DOI:** 10.3389/fped.2023.1234964

**Published:** 2023-10-06

**Authors:** Alison Protain, Kimberly Firestone, Saima Hussain, Daniel Lubarsky, Howard Stein

**Affiliations:** ^1^ Pediatrix Medical Group Akron Ohio, Akron Children’s Hospital, Akron, OH, United States; ^2^ Department of Neonatology, Akron Children’s Hospital, Akron, OH, United States; ^3^ Department of Pediatrics, ProMedica Russell J. Ebeid Children’s Hospital, Toledo, OH, United States; ^4^ Department of Pediatrics, Monroe Carell Jr. Children’s Hospital at Vanderbilt, Nashville, TN, United States; ^5^ Northwest Ohio Neonatal Associates, ProMedica Russell J. Ebeid Children’s Hospital, Toledo, OH, United States

**Keywords:** neurally adjusted ventilatory assist (NAVA), premature neonates, apnea of prematurity (AOP), continuous positive airway pressure (CPAP), NAVA-PAP

## Abstract

**Background:**

Neonates with apnea of prematurity (AOP) clinically deteriorate because continuous positive airway pressure (CPAP) provides inadequate support during apnea. Neurally adjusted ventilatory assist (NAVA) provides proportional ventilator support from the electrical activity of the diaphragm. When the NAVA level is 0 cmH_2_O/mcV (NAVA-PAP), patients receive CPAP when breathing and backup ventilation when apneic. This study evaluates NAVA-PAP and time spent in backup ventilation.

**Methods:**

This was a prospective, two-center, observational study of preterm neonates on NAVA-PAP for AOP. Ventilator data were downloaded after 24 h. The number of clinically significant events (CSEs) was collected. A paired *t*-test was used to perform statistical analysis.

**Results:**

The study was conducted on 28 patients with a gestational age of 25 ± 1.8 weeks and a study age of 28 ± 23 days. The number of CSEs was 4 ± 4.39/24 h. The patients were on NAVA-PAP for approximately 90%/min, switched to backup mode 2.5 ± 1.1 times/min, and spent 10.6 ± 7.2% in backup.

**Conclusion:**

Preterm neonates on NAVA-PAP had few CSEs with minimal time in backup ventilation.

## 1. Introduction

Premature neonates remain vulnerable to the physiologic consequences of apnea of prematurity (AOP). Periods of clinical deterioration characterized by bradycardia and desaturation, referred to as clinically significant events (CSEs), are frequently encountered in the neonatal intensive care unit (NICU) (1). Caffeine citrate and continuous positive airway pressure (CPAP) are commonly used therapeutic modalities that demonstrate significant benefits (2–4). Unfortunately, increasing respiratory support may be required for the smallest and most fragile premature neonates. Non-invasive respiratory strategies have shown promise, with synchronization of nasal ventilation showing further improvement for respiratory stability (5), although intubation and mechanical ventilation may be needed for severe apnea (2, 3, 6).

**Figure 1:**
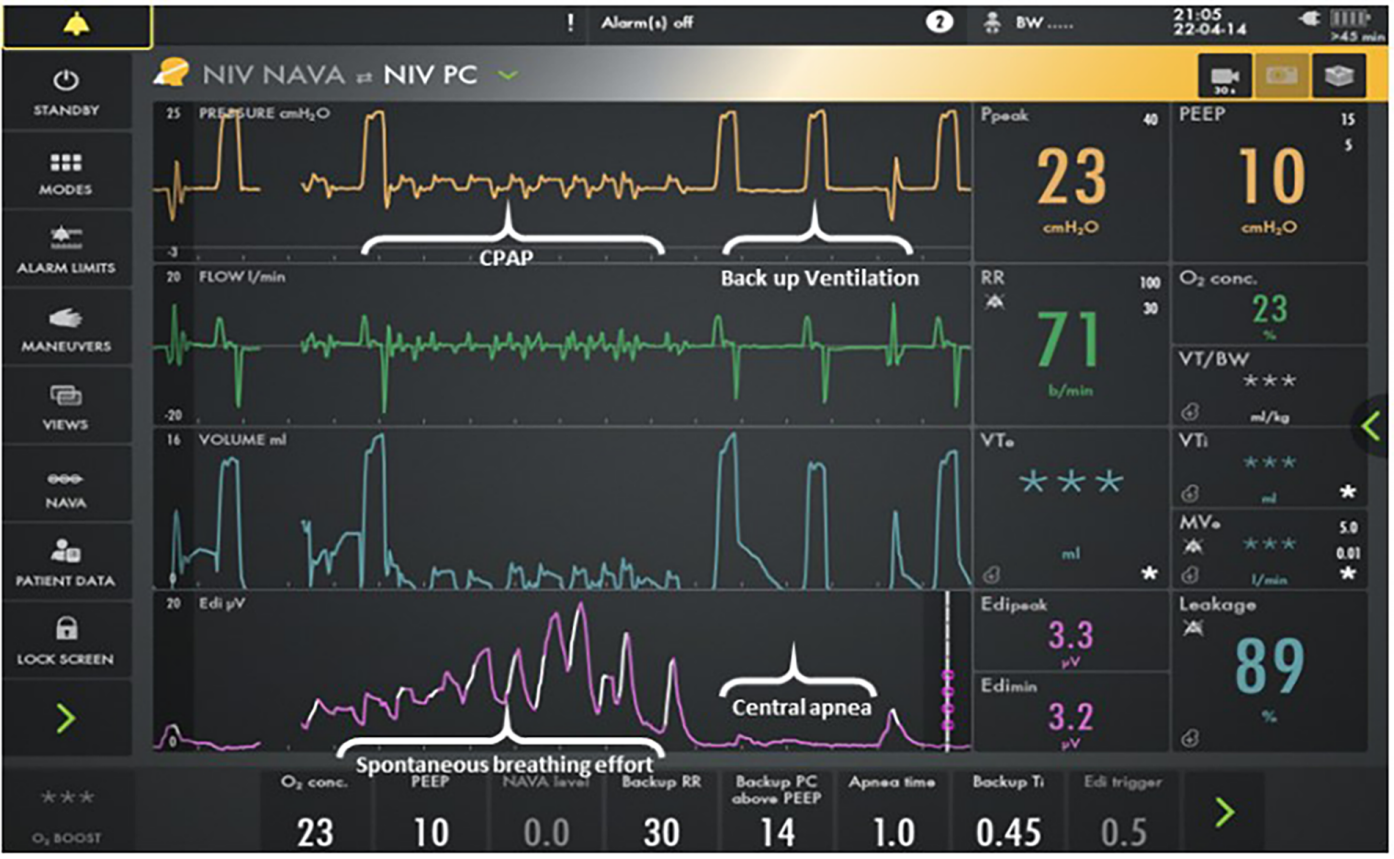
Screenshot for the Servo-I ventilator. The bottom line represents the Edi signal. The next lineup is volume, and the third lineup is flow. The top line is pressure. Patient interface resistance compensation in the NAVA software program provides minimal support above CPAP when breathing. Backup ventilation is provided when apneic. Auto-triggering does not occur as neural triggering is the mechanism for initiation.

Neurally adjusted ventilatory assist (NAVA) provides support in synchrony with the respiratory efforts of a patient based on the detected electrical activity of the diaphragm (Edi). It is delivered with the Servo-I/U/N ventilator (Getinge, Germany) using NAVA software. The NAVA level is a proportionality factor that converts the Edi signal into a pressure above the positive end-expiratory pressure (PEEP) supporting each spontaneous breath. If no Edi signal is detected for a predetermined amount of time (apnea time), the ventilator switches into pressure control ventilation until the patient breathes spontaneously again, which provides a minimum rate (7). If the NAVA level is set at 0 cmH_2_O/mcV, the patient receives minimal synchronized support above the baseline PEEP while breathing (CPAP) and backup ventilation when apneic (Figure 1).

Although NAVA ventilation has been in use for over a decade, only one previous study has shown a statistically significant reduction in CSEs for premature neonates ventilated on non-invasive NAVA of 0 (NAVA-PAP) vs. CPAP via the RAM cannula (1). However, the mechanism for this improvement was not studied. This study aims to evaluate NAVA-PAP acting as CPAP with backup ventilation further. The primary outcomes focus on the time in NAVA vs. backup ventilation and delineating ventilator pressures and respiratory rates (RRs).

## 2. Materials and methods

This was a prospective, two-center, observational study of preterm patients admitted to the NICUs of ProMedica Ebeid Children's Hospital and Akron Children's Hospital during the period between August 2019 and February 2020. The patients were placed on NAVA-PAP for AOP at the treating provider's discretion. IRB approval and informed consent were obtained. Akron Children's Hospital Institutional Review Board and ProMedica IRB reviewed and approved the studies involving human/animal participants. The patients/participants (legal guardian/next of kin) provided written informed consent to participate in this study. All research was conducted in accordance with the ethical standards of all applicable national and institutional committees and the World Medical Association's Helsinki Declaration. Ventilator data were downloaded after 24 h of using NAVA-PAP. Each data point was the breath-to-breath data averaged over 1 min. Therefore, 1,440 data points were collected for each subject. The demographics and number of CSEs were collected. CSEs were defined as bradycardia (less than 80 beats/min) or desaturation (less than 90%) lasting more than 10 s. CSEs were retrospectively collected from the electronic health record during the data collection on NAVA-PAP. A paired *t*-test was used to perform statistical analysis.

## 3. Results

**Figure 2:**
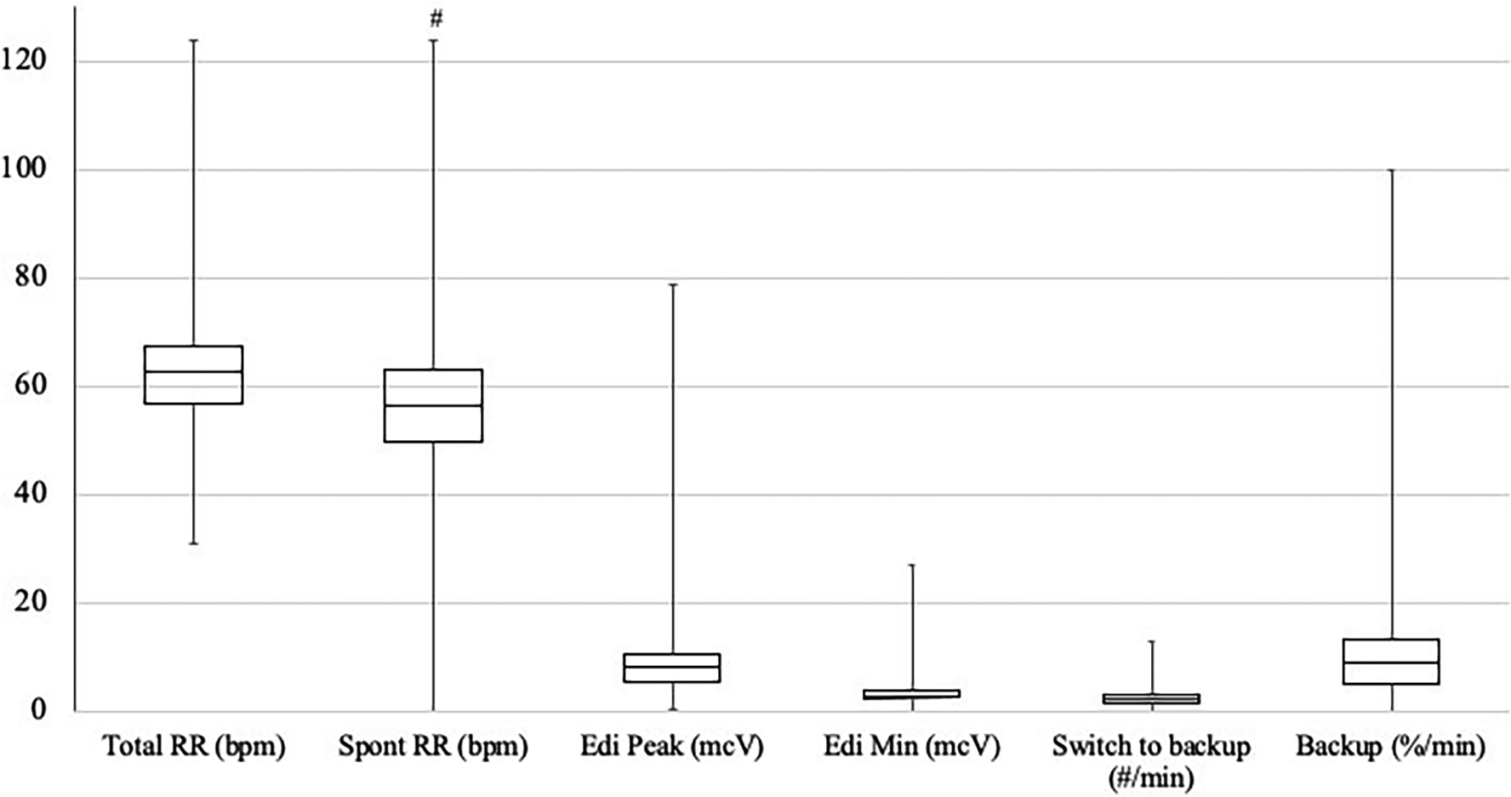
Distribution of RR, Edi, and switches to backup. Units are noted in parentheses following each variable. The box plots show the median and first and third quartiles. The whiskers are the minimum and maximum values. ^#^*p* < 0.05 compared with total RR.

**Figure 3:**
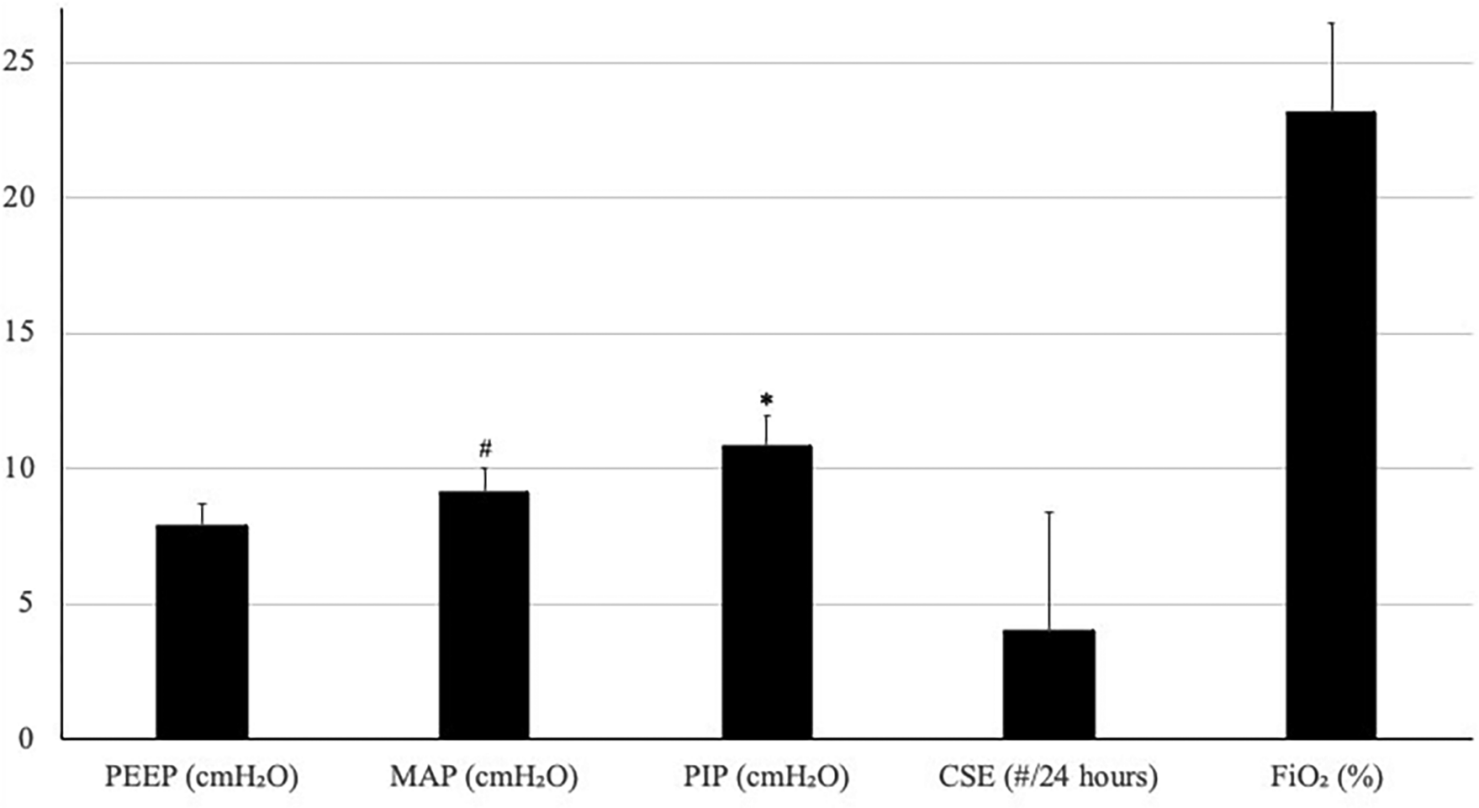
Average (SD) of PEEP, MAP, PIP, CSE, and FiO_2_. Units are noted in parentheses following each variable. ^#^*p* < 0.05 compared with PEEP, *p < 0.05 compared with MAP.

A total of 28 patients on NAVA-PAP were enrolled. In total, 40,320 data points (each data point = 1 averaged minute) were collected for the 28 patients. All patients experienced AOP, were treated with caffeine, and were on the RAM cannula nasal interface. Table 1 shows the demographics of the patients. Ventilator settings were a NAVA level of 0, an apnea time of 2 s (minimum rate of 30 breaths per minute), a peak inspiratory pressure (PIP) limit of 35–40 cmH_2_O, a PEEP of 7.9 ± 0.8 cmH_2_O, backup settings of PIP 19 ± 3.6 cmH_2_O,, and a rate of 43 ± 5.48. Patients were in NAVA-PAP for approximately 54 s per minute, went into the backup ventilation mode 2.5 ± 1.1 times per minute, and spent approximately 6 s of each minute in the backup mode (Figure 2). This calculates to an average of 150 brief switches per hour for a collective total of 6 min/h in the backup mode. The total RR was slightly higher than the spontaneous RR. Figure 3 shows that the number of CSEs was 4 ± 4.39/24 h. The mean airway pressure (MAP) was higher than the measured PEEP, and the average PIP was higher than MAP. The RAM nasal interface had a 91.9 ± 4.5% leak, with a mean supplemental oxygen requirement of 23.2 ± 3.3%.

**Table 1:** Demographics.

Number of patients	28 (female 57%)
Gestational age (weeks)	25 ± 1.8 (range 23–29)
Birth weight (g)	860 ± 257 (range 460–1,100)
Median Apgar scores	5 (1 min) 8 (5 min)
Prenatal steroids	79%
Surfactant	92%
Age at study (days)	28 ± 23 (range 3–91)
Weight at study	1,077 ± 375 (range 405–2,270)
IVH (grades III–IV)	0%
NEC	3.5%
ROP	14%
CLD	17%

IVH, intraventricular hemorrhage; NEC, necrotizing enterocolitis; ROP, retinopathy of prematurity; CLD, chronic lung disease.

Demographics of the study patients. Ages and weights are expressed as average ± SD.

## 4. Discussion

The expanded use of non-invasive ventilation strategies to decrease the need for intubation and invasive ventilation remains a high priority (8). CPAP still remains the gold standard, although a significant percentage of preterm neonates will require increased respiratory support (6, 9, 10). Non-invasive respiratory support subsequently evolved with options such as nasal intermittent positive pressure ventilation (NIPPV) to augment lung inflation and respiratory muscle unloading (11). The flow-triggered synchronized mode of NIPPV was also found to have beneficial effects on reducing apnea and desaturations compared with CPAP and NIPPV (12).

NAVA, a novel mode of ventilation, changed the paradigm by providing synchronized ventilation in which the patient controls both timing and degree of ventilatory assistance. Several studies have demonstrated decreased PIPs, oxygen requirement, and apnea in preterm neonates receiving NAVA ventilation compared with those receiving traditional synchronized intermittent mechanical ventilation (SIMV) and pressure control ventilation (13–16).

Compared with CPAP, some data suggest that NIV NAVA in neonates may reduce the need for intubation, facilitate early extubation, and decrease extubation failures (17–19). NIV NAVA has also been shown to decrease the number of CSEs compared with non-synchronous, non-invasive ventilation (20).

NAVA-PAP is the only non-invasive mode that delivers CPAP while breathing and backup ventilation when the patient is apneic. Utilizing NAVA-PAP as a strategy in neonates with AOP while on CPAP was recently studied and demonstrated significant benefits in reducing CSEs (1). This approach offers the advantage of reducing CSEs while minimizing exposure to non-invasive ventilation.

When NIV NAVA is set at 0 cmH_2_O/mcV, the Servo-I/U delivers a PIP of 2 cmH_2_O above PEEP in synchrony with the spontaneous breaths of the patient (1). NAVA software has patient interface resistance compensation and accounts for this small increase in pressure observed above PEEP. Buzzella et al. (21) previously demonstrated that higher CPAP levels resulted in fewer extubation failures. Owen et al. (22) showed that NIPPV did not deliver any measurable tidal volume during periods of apnea. Therefore, it is possible that the minimally augmented pressure delivered with each breath translating into a higher overall MAP is responsible for the improvement noted in CSEs in previous studies (1) but could also be related to the stimulation of the patient's respiratory drive during backup ventilation.

Even though the average set PIP delivered 19 cmH_2_O with each breath, the measured average PIP was only 10.9 cmH_2_O. Since there was no proximal sensor in use at the nasal interface, this decrease in the overall average measured PIP was expected considering that there were so few set backup breaths delivered each minute. The average PIP was higher than MAP (10.9 vs. 9.1 cmH_2_O). This was because the intermittent backup breaths delivered occur only during backup ventilation and did not deliver any additional pressure with any spontaneous breaths. Both Edi peak and minimum were within previously described ranges for preterm neonates (23).

Our data demonstrate that the time preterm neonates spent in backup ventilation was quite low. Studies have shown improved patient–ventilator synchrony with NAVA ventilation, with a resultant decrease in the number of apneic events (14, 20). Although these studies attributed the decreased CSEs to being on NIV NAVA, perhaps it is the additional breaths delivered during periods of apnea that contributed to the decrease in the number of CSEs. These additional backup breaths account for the total RR higher than the spontaneous RR (64 vs. 58 breaths per minute) but remains well within the normal RR range for neonates. Using an apnea time of 2 s meant that all patients had a minimum rate of 30 breaths per minute. Because of this short apnea time, there were multiple brief switches to backup pressure control ventilation, but the percentage of time spent in the backup mode was low. This short apnea time may have prevented any extended periods of apnea and contributed to the improvement in CSEs, as supported by a previous pilot trial that demonstrated that short apnea times should be utilized for neonatal patients ventilated with NIV NAVA to promote clinical stability and decrease the occurrence of clinically important events (24).

This study is limited by several factors. This was a descriptive study, so no control group was needed. Reliance was on bedside charting to determine the number of CSEs. In addition, there was an inability to determine the exact mechanism for the CSE reduction reported by a previous study. Prospective randomized controlled studies are needed to further explore the reduction in CSEs and other potential benefits of NAVA-PAP. This study only included neonates on the RAM cannula interface, which limits the generalizability of the findings to other nasal interfaces. However, useful data were provided to support further investigation of NAVA-PAP for treatment of preterm neonates with AOP.

In conclusion, NAVA-PAP in preterm neonates acted as minimally augmented CPAP during spontaneous ventilation and provided backup ventilation when apneic. Increased MAP and total RR may explain why this mode was effective. Despite the large leak, the RAM nasal interface was effective at delivering CPAP (NAVA level of 0) with backup ventilation. This modality may offer a safe and effective option to avoid excessive non-invasive ventilation or intubation in premature neonates failing CPAP because of frequent CSEs.

## Data Availability

The raw data supporting the conclusions of this article will be made available by the authors, without undue reservation.
